# Antonie van Leeuwenhoek and the dawn of microscopic observation: a narrative review from Delft’s lens to the modern microscope

**DOI:** 10.1186/s42649-026-00123-z

**Published:** 2026-02-02

**Authors:** Hyunjeong Lee, Im Joo Rhyu

**Affiliations:** 1 Department of Anatomy, Korea University College of Medicine, Goryeodae-ro 73 (Anam-dong 5ga), Seongbuk-gu, Seoul, 02841 Korea; 2 GERONMED, Co. Ltd, 117-3Hoegi-ro Dongdaemun-gu, Seoul, 02455 Korea

**Keywords:** Microscopy/history, Microscopes/standards, Optics, Microorganisms/physiology, Blood Cells/ultrastructure, Spermatozoa/physiology, Microscopes/single-lens design, Insecta/anatomy & histology, Crystallography/microscopy

## Abstract

Antonie van Leeuwenhoek (1632–1723) transformed observation into science through the power of a single handmade lens. His work emerged from the visual culture of seventeenth-century Delft, where craftsmanship, optics, and artistic precision intersected. While Robert Hooke’s compound microscope introduced the idea of microscopic visualization, Leeuwenhoek’s single-lens instruments achieved far superior magnification and resolution by minimizing optical interfaces. Using these deceptively simple devices, he documented the first observations of free-living microorganisms, fungal hyphae, red blood cells, capillary flow, oral bacteria, and spermatozoa in more than two hundred letters to the Royal Society of London.

But his investigations reached far beyond microbiology. Leeuwenhoek also examined the barbed structure of the bee sting, the ordered vessels of ash wood, and the geometric microstructure of crystals and salts—demonstrating that hidden organization pervades both living and non-living matter. These studies established microscopy as a universal investigative tool, capable of unifying biology, medicine, botany, and early materials science under a single optical principle.

Leeuwenhoek’s work marks one of the earliest examples of how rigorous observation can redefine scientific domains. His use of a home-crafted single lens created an empirical foundation for biological microscopy that persists to this day. The legacy of his minimalist optical design also survives in the digital age: modern clip-on smartphone microscopes and paper-based platforms such as the Foldscope reproduce the same single-lens principle through micro-optics mounted directly onto digital sensors.

Three and a half centuries later, his work continues to remind us that new worlds do not emerge from new theories alone, but from new ways of seeing.

## Through the artist’s window

**Fig. 1:**
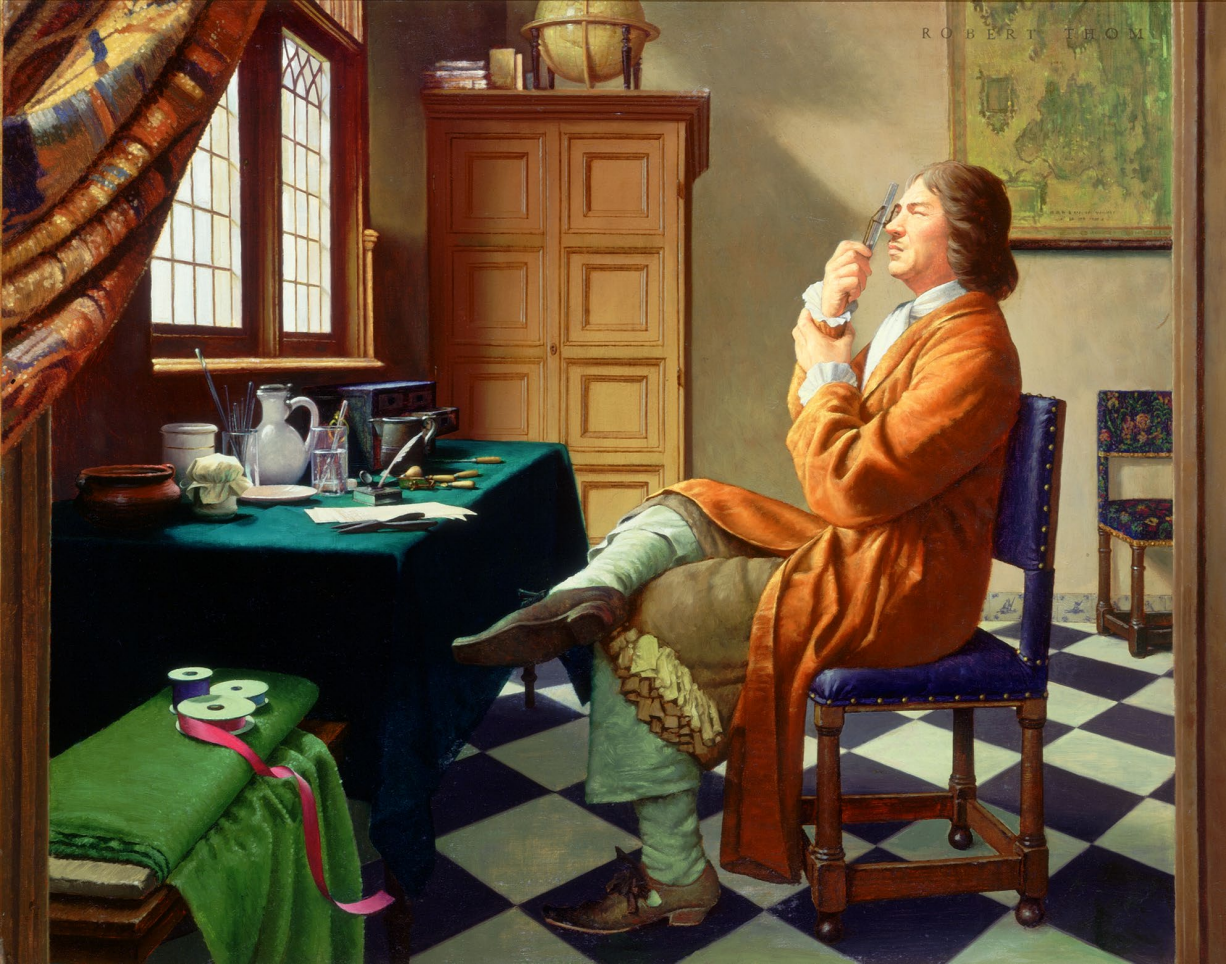
Leeuwenhoek at work — artistic interpretation of early microscopy. Painting by Robert Thom from the Great Moments in Medicine series. Courtesy of the University of Michigan, used with permission

The story of microscopy begins not in a laboratory but in the quiet, light-filled rooms of seventeenth-century Delft. Robert Thom’s modern painting of Antonie van Leeuwenhoek at work (Fig. 1) captures the essence of that world: a modest workshop bathed in northern light, where glass, brass, and curiosity meet. Here a cloth merchant polishes a tiny bead of glass, unaware that it will become the first portal to the invisible. Around him, Delft’s merchants and artisans exchanged instruments and ideas—the same city that inspired the painter Johannes Vermeer to study how light defines form.

Vermeer’s The Geographer (1668) and The Art of Painting (1666–1668) portray figures surrounded by maps, globes, and measuring instruments, immersed in observation. Leeuwenhoek and Vermeer likely walked the same narrow streets, and archival notarial records indicate that Leeuwenhoek served as the executor of Vermeer’s estate after the painter’s death (Lens on Leeuwenhoek n.d.). Both shared a fascination with precision: Vermeer captured the geometry of light upon pigment, while Leeuwenhoek captured its refraction through glass. In their hands, light was both subject and tool.

This artistic context frames the birth of scientific microscopy. The seventeenth century witnessed the convergence of optics, craftsmanship, and empirical curiosity. Within that milieu, Leeuwenhoek’s single-lens microscope emerged not merely as a technical device but as a product of the same Dutch visual culture that prized clarity, material fidelity, and truth to observation (Dobell 1932; Schierbeek 1959). As Vermeer turned the ordinary into revelation on canvas, Leeuwenhoek transformed a droplet of water into a universe of motion. The two perspectives—artistic and scientific—are separated by medium but united by their pursuit of seeing more clearly. This convergence created the cultural and intellectual environment in which early microscopy could flourish.

## Compound vs. single-lens microscopes: an optical comparison

**Fig. 2:**
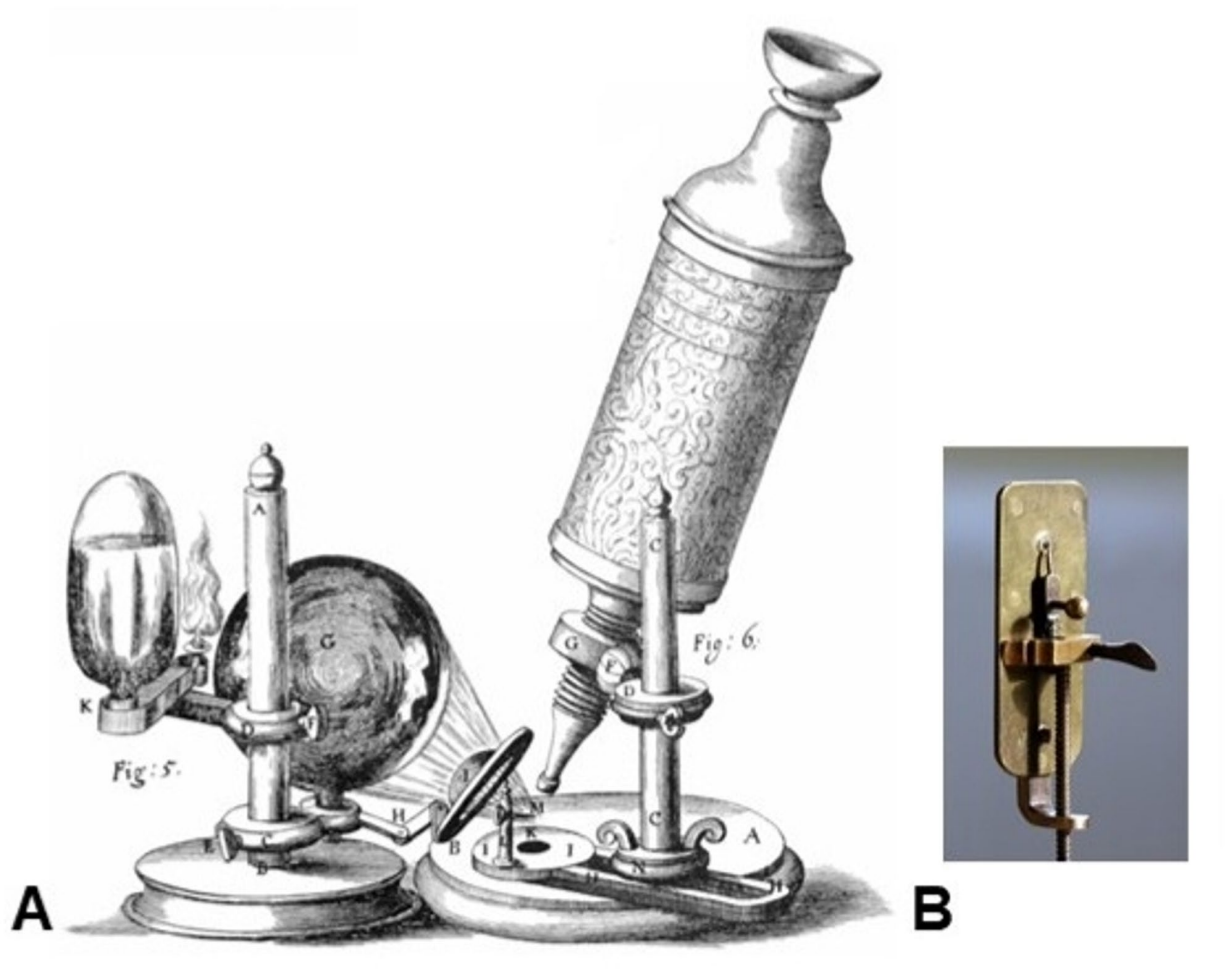
Early microscopes of the seventeenth century. **A** Robert Hooke’s compound microscope, as illustrated in Micrographia (1665). Public domain, originally published by the Royal Society of London. **B** Replica of Antonie van Leeuwenhoek’s single-lens microscope. Image by Jeroen Rouwkema, sourced from Flickr (https://www.flickr.com/photos/rouwkema/2262158965/), licensed under CC BY-SA 3.0

When Robert Hooke published Micrographia in 1665, the compound microscope had already entered scientific fashion (Hooke 1665). It employed two or more lenses arranged in tandem to magnify an image—an ingenious but imperfect solution. Each glass surface introduced refraction errors, and with seventeenth-century glass quality and polishing techniques, chromatic and spherical aberrations produced colored fringes and blurred edges (Gest 2004; Dobell 1932; Lane 2015). Hooke’s instrument—illustrated in Micrographia and shown in Fig. 2A—was capable of about 30–50× magnification, but often sacrificed clarity for scale (Gest 2004). Yet Micrographia was revolutionary in concept: it revealed that unseen worlds existed and could be drawn with scientific precision (Hooke 1665; Gest 2004).

Leeuwenhoek approached the same problem from the opposite direction. Rather than multiply lenses, he eliminated all but one. By grinding a minute glass sphere—sometimes no larger than a pinhead—and mounted it between brass plates with adjustable screws, he reduced optical interfaces to a minimum (Dobell 1932; Zuidervaart 2016). His single-lens instrument—represented in Fig. 2B—achieved magnifications far exceeding those of contemporary compound microscopes. Surviving examples of his microscopes, now preserved in Delft and London, show magnifications up to approximately 266× with exceptional contrast (Zuidervaart 2016; van Delft et al. 2018; Lane 2015). Modern neutron tomography and interferometric analyses confirm that the tiny single lenses he fashioned possessed nearly ideal spherical curvature and minimal inhomogeneity for their size (van Delft et al. 2018; Zuidervaart 2016).

These differences are summarized in Table 1, which compares the optical and mechanical characteristics of early compound microscopes with those of Leeuwenhoek’s single-lens instruments (Hooke 1665; Dobell 1932; Gest 2004; Zuidervaart 2016; van Delft et al. 2018; Lane 2015). Hooke’s multi-lens system provided a wider field of view and more comfortable working distance, but it suffered greatly from chromatic and spherical aberration, unstable illumination, low brightness, and limited resolving power (Hooke 1665; Gest 2004; Lane 2015). By contrast, Leeuwenhoek’s single spherical lens achieved magnifications up to four times higher, with resolving powers of 1–2 μm—far superior to the 5–10 μm resolution typical of compound microscopes of the period (Zuidervaart 2016; van Delft et al. 2018; Lane 2015). His minimalist design minimized internal reflections, maximized brightness, and enabled the discovery of bacteria, spermatozoa, red blood cells, and capillaries—structures that the compound microscope of his time could not reveal (Dobell 1932; Gest 2004).

**Table 1:** Optical and mechanical differences between hooke’s compound microscope and leeuwenhoek’s single-lens microscope

Feature	Early Compound Microscope (Hooke, 1660s)	Early Single-Lens Microscope (Leeuwenhoek, 1670s)	Key References
Optical design	Two or more lenses in series (objective + eyepiece)	Single spherical glass bead lens	Hooke (1665); Zuidervaart (2016)
Magnification	~ 30–50×	68× to 266× (based on surviving lenses)	van Delft et al. (2018); Gest (2004)
Resolution	5–10 μm, limited by optical aberrations	1–2 μm, highest of the period	van Delft et al. (2018); Lane (2015)
Aberration	Severe chromatic/spherical aberration	Minimal chromatic and spherical aberration	Dobell (1932); Gest (2004)
Image brightness	Low brightness; depended on mirrors and candles	High brightness (minimal light loss)	Hooke (1665)
Field of view	Relatively wide	Very narrow	Zuidervaart (2016)
Sample handling	Early stage/slip mounting	Specimen mounted directly on a pin	Gest (2004)
Illumination	Mirror + candle or oil lamp; unstable illumination	Direct sunlight or reflected daylight	Hooke (1665)
Lens fabrication	Ground and polished multi-element lenses	Melted glass bead formed at tip of a rod	van Delft et al. (2018)
Scientific impact	Enabled structural studies in insects and plants; foundation of Micrographia	Enabled discovery of bacteria, spermatozoa, red blood cells, capillaries	Dobell (1932); Gest (2004)
Ease of use	More comfortable viewing distance	Eye must be within 1–3 mm of lens; difficult and tiring	Dobell (1932)
Mechanical design	Larger, bulkier body with complex focusing system	Small, pocket-sized (3–5 cm); brass plates and two screws	Zuidervaart (2016)

Despite the inconvenience of having to place the eye within millimetres of the lens, the simplicity of optical geometry gave the single-lens microscope a decisive advantage that persisted well into the nineteenth century, until achromatic doublets and corrected objectives finally overcame compound aberrations (Dobell 1932; Lane 2015). In effect, Leeuwenhoek had discovered—empirically rather than theoretically—the principle later formalized by Abbe: resolution depends as much on the purity and quality of the optical path as on magnification itself (Abbe 1873). His instruments embodied a paradox that continues to guide microscope design today: the fewer the imperfections between the observer and the object, the deeper the truth that can be seen. This empirical insight later anticipated the formalization of resolution limits that became central to modern optical theory.

## Key reports of Leeuwenhoek

**Fig. 3:**
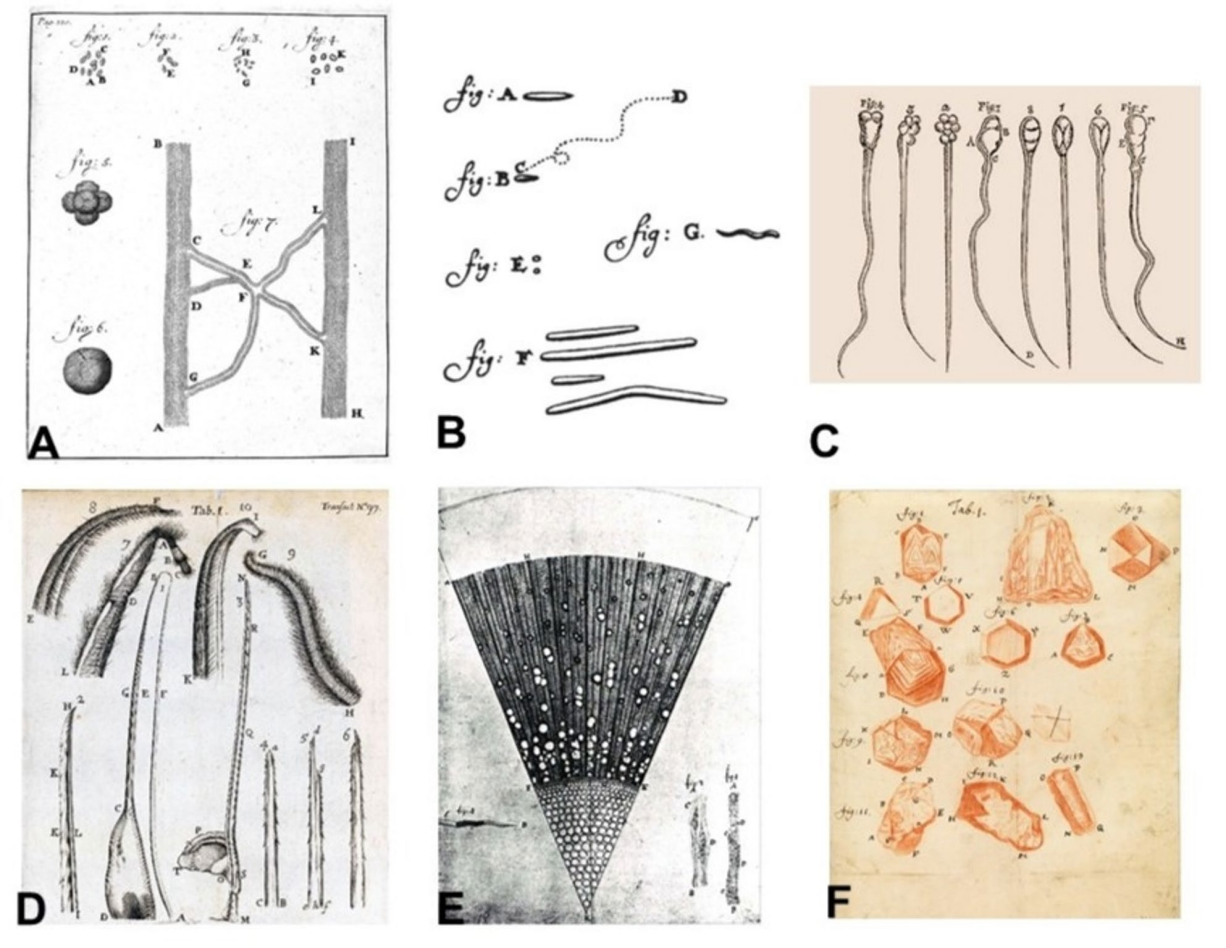
Representative drawings from Antoni van Leeuwenhoek’s pioneering microscopic observations, all from works now in the public domain. **A** Early depictions of blood and capillary connections, including some of the first visualizations of red blood cells. **B** Morphological varieties of microorganisms (“animalcules”) observed in water, dental plaque, and fermented liquids, illustrating the earliest descriptions of bacteria. **C** Animal spermatozoa observed by Leeuwenhoek, representing the earliest detailed descriptions of reproductive cells. **D** Structure of the bee stinger and its barbed apparatus, representing one of the earliest functional anatomical observations. **E** Transverse sections of wood and plant tissues, revealing vessel patterns and microscopic organization. **F** Geometric forms of crystals and mineral structures, documenting Leeuwenhoek’s studies of inorganic microscopic morphology

Once the single-lens microscope reached its mature form, Leeuwenhoek turned his instrument from glass to life itself. Between 1673 and 1723 he sent more than two hundred letters to the Royal Society of London, each a careful record of experiments performed at his workbench in Delft (Royal Society of London 1675–1684; Dobell 1932; Gest 2004). The progression of these discoveries—from water organisms to blood, bacteria, spermatozoa, plant tissues, insects, and crystalline materials—is summarized in Table 2 and illustrated in Fig. 3A–F.

**Table 2:** Representative specimens observed by Antonie Van Leeuwenhoek

Category / Specimen type	Example and description	Year / Letter	Scientific significance	Representative references
Fresh and rain water	Rain, pond, and well water with animalcules of various forms	1674–1676	First observation of free-living microorganisms	van Leeuwenhoek (1677)
Pepper-water infusion	Water infused with ground pepper, left to stand	1676	Demonstrated spontaneous microbial growth	van Leeuwenhoek (1677); Gest (2004)
Fungi and molds	Mold filaments and spores from decaying plants	1673–1675	First microscopic description of fungal hyphae	Dobell (1932)
Plant and insect tissues	Wood fibers, bee stings, insect mouthparts	1675–1680	Early histological observations	van Leeuwenhoek (1675); Schierbeek (1959)
Animal blood and capillaries	Human, frog, eel blood; capillary flow	1674–1680	Visual confirmation of microcirculation	van Leeuwenhoek (1674)
Oral bacteria	Dental plaque with motile microorganisms	1683	First bacterial observation and classification	van Leeuwenhoek (1684)
Spermatozoa	Motile filaments in semen	1677	First description of sperm cells	van Leeuwenhoek (1677)
Crystals and minerals	Salt crystals, textile fibers	1670s	Observation of geometric structure	Lens on Leeuwenhoek database

### Water and microorganisms

In 1674, he examined pond and rainwater and found animalcules of astonishing variety—coiled, rod-shaped, and spiral forms that swam and divided. This was the first discovery of free-living microscopic life (Royal Society of London 1675–1684; Dobell 1932; Gest 2004).

### Fungi and molds

His early correspondence included descriptions of molds and spores, offering the earliest microscopic depiction of fungal hyphae (Dobell 1932; Schierbeek 1959). Leeuwenhoek observed filamentous structures arising from decaying plant material and bread mold, noting their branching patterns and the presence of round or oval reproductive bodies. These observations broadened the emerging microscopic world beyond protozoa and bacteria, demonstrating that multicellular organisms—such as fungi—also possessed hidden architectures.

### Blood and microcirculation

In 1674 he described blood as composed of “very small red globules, flat like a coin and somewhat hollow in the middle,” providing the earliest recognizable representation of the biconcave red blood cell. His drawing of microvascular blood flow (Fig. 3A) demonstrated continuous capillary circulation (Royal Society of London 1675–1684; Dobell 1932; Gest 2004). Recent reassessment suggests that he examined both vertebrate erythrocytes and arthropod hemocytes over more than thirty letters (Davis 2022).

### Bacteria and protozoa

In 1683 he examined dental plaque and observed “exceeding small creatures moving very prettily.” He differentiated bacteria into spherical, rod-shaped, and spiral forms—centuries before modern taxonomy (Fig. 3B). This remains a foundational milestone in microbiology (Gest 2004; Lane 2015).

### Spermatozoa

In 1677 he visualized motile filaments in human and animal semen (Fig. 3C), cautiously calling them “animalcules.” His identification of spermatozoa shifted debates on generation toward cellular mechanisms of fertilization (Royal Society of London 1675–1684; Dobell 1932).

### Insect anatomy and the mechanism of stinging

His drawings of the bee stinger and its barbed apparatus (Fig. 3D) revealed the structural basis of envenomation, representing one of the earliest examples of functional anatomical microscopy (Dobell 1932; Schierbeek 1959).

### Plant anatomy and wood microstructure

His examination of ash wood (Fraxinus) showed the ordered arrangement of vessels and fibers within the plant stem (Fig. 3E). These observations anticipated later developments in plant histology and demonstrated that plant tissues, like animal tissues, are built from repeated structural units (Dobell 1932; Lane 2015).

### Crystals, salts, and mineral microstructure

Leeuwenhoek also investigated inorganic materials, sketching the geometric forms of crystals and salts (Fig. 3F). These studies represent some of the earliest microscopic analyses of crystalline structure, extending microscopy beyond biology toward early materials science (Dobell 1932).

## Historical significance and summary

Leeuwenhoek’s investigations transformed curiosity into method. By constructing his own lenses and systematically recording what he saw, he established the foundations of experimental microscopy (Dobell 1932; Gest 2004). His letters to the Royal Society of London served as reproducible visual evidence—a new standard for observational science (Royal Society of London 1675–1684). His discoveries of red blood cells, bacteria, spermatozoa, and capillary circulation demonstrated that biological organization exists across multiple scales, linking anatomy, physiology, and microbiology into a coherent microscopic framework (Dobell 1932; Schierbeek 1959; Davis 2022).

Yet the scope of his work extended far beyond microorganisms and human physiology. He applied his single-lens microscope to insects, plants, and inorganic materials, thereby pioneering not only biological microscopy but also botanical anatomy and the earliest forms of materials science. His observations of blood, microbes, spermatozoa, insect stingers, plant vessels, and crystalline structures (Fig. 3A–F) revealed that the microscopic world encompasses structural order across both living and non-living systems. Taken together, these investigations show that Leeuwenhoek established microscopy as a universal investigative tool—one capable of unifying biology, medicine, and materials science under a single optical principle (Dobell 1932; Gest 2004; Davis 2022).

Optically, his instruments embodied an empirical solution to the problem of resolution. By minimizing optical interfaces, he achieved clarity that contemporary compound microscopes could not match (Zuidervaart 2016; van Delft et al. 2018; Lane 2015). This minimalist approach anticipated later innovations in high-resolution microscopy—from Abbe’s diffraction theory and phase-contrast imaging to super-resolution techniques such as STORM, PALM, and STED, and ultimately cryo-electron microscopy, which now visualize biomolecules at near-atomic scale (Abbe 1873; Lane 2015). The same single-lens principle survives today in clip-on smartphone microscopes and in origami-based devices such as the Foldscope, where a glass-bead lens is paired directly with a digital sensor to deliver Leeuwenhoek-level magnification in classrooms and in the field (Cybulski et al. 2014).

His microscopes remind us that progress in imaging has always depended as much on the hands that shape glass as on the minds that interpret its light. In the twenty-first century, when optical systems are complemented—and sometimes replaced—by detectors, algorithms, and computational reconstruction, the same principle persists: new worlds appear whenever we find new ways to see. From insect stingers to plant vessels and mineral crystals, Leeuwenhoek showed that the natural world—organic and inorganic—possesses hidden structures that can be revealed through even the simplest optical tools. The continuum from his handmade lenses to modern optical and computational microscopy stands as a testament to an enduring truth: the frontier of discovery begins with the act of looking more closely.

## Data Availability

Not applicable.
